# Personal experience in transnasal endoscopic resection of the olfactory groove meningiomas. What can an otolaryngologist offer to a neurosurgeon?

**DOI:** 10.1007/s00405-013-2645-3

**Published:** 2013-08-02

**Authors:** Andrzej Skorek, Wiesław Liczbik, Czesław Stankiewicz, Wojciech Kloc, Łukasz Plichta

**Affiliations:** 1 Otolaryngology Department, Medical University in Gdańsk, Gdańsk, Poland; 2 Neurosurgical Department, Pomeranian Traumatology Centre in Gdańsk, Gdańsk, Poland

**Keywords:** Meningioma olfactory groove, Transnasal endoscopic approach

## Abstract

Olfactory groove meningioma is a demanding therapeutic problem involving two medical specialties, otolaryngology and neurosurgery. The use of transnasal endoscopic (TNE) approach to the tumour has been proved effective in many publications. Three patients with meningiomas localized in olfactory groove were treated in 2011 and 2012 by the otolaryngologist-neurosurgeon team using TNE approach and neuronavigation. The diagnosis was based on MR and CT images. In all patients after tumour removal an endoscopic anterior cranial fossa floor reconstruction was performed using homogeneous cartilage or titanium mesh and Hadad-Bassagasteguy flap. During postoperative period in all patients lumbar drainage was used. There were no cerebrospinal fluid leakage episodes. No recurrence was observed in 22, 12 and 8 months of follow-up, respectively. The authors describe otolaryngological and neurosurgical aspects of TNE approach to anterior cranial fossa with special regard to possible radical resection (according to Simpson) and reconstruction of the bony postoperative defect. TNE is a feasible operative method in olfactory groove meningioma management due to good tumour visibility, lack of brain traction, limited neurovascular structure manipulation and acceptable risk of neurological deficiencies when compared to open approach. Cosmetic aspect and short hospitalization is also of great importance.

## Introduction

Meningiomas are benign tumours of the central nervous system originating in meningothelial cap cells of arachnoid tuberculations and characterized by diverse growth dynamics. Cerebral falx region, cranial vault, sphenoid bone wings, perisellar region and olfactory groove are the most common primary locations for this tumour. Adults between 40 and 60 are most likely to be diagnosed with meningioma with higher prevalence in females when compared to males. Primary location implies occurrence of certain symptoms, as well as, the method of treatment [1, 2, 8, 9].

Localized inside olfactory groove, meningiomas are scarcely symptomatic or present with non-specific, common symptoms (e.g. headache) or even symptoms overlooked by the patients (unilateral anosmia). Progress in tumour size implies more serious disturbances connected with impression on optic nerves or chiasm (visual impairment), frontal lobe (personality disorders, memory disturbances) or seizures [2, 9].

Typical treatment entails surgical resection via external approach: frontal/bifrontal, pterional [2, 8, 9]. The development of endoscopic transnasal surgical techniques, especially dynamic at the turn of the twentieth and twenty first centuries, emboldened many surgeons to introduce this microinvasive and relatively safe method for operating in the region of the anterior cranial fossa under and over the cribriform plate of the ethmoid bone [9]. As a result of the cooperation between two specialists, an otolaryngologist and a neurosurgeon, certain borders may be now crossed without fear.

In the available literature we have found only three papers describing series of patients with tumours of such location treated with transnasal endoscopic approach (TNE) (15, 8, 4 cases). Taking into consideration small number of these reports we would like to share our experience in this method of treatment.

## Materials and methods

**Fig. 1:**
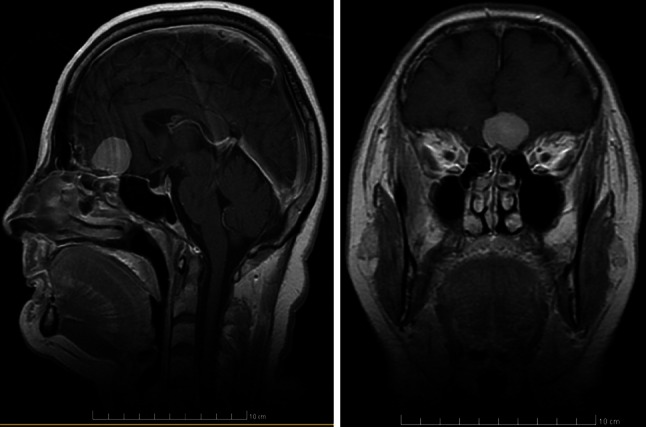
MRI (T2). Female patient aged 52—case number 1, history of anosmia for 1 year, seizures for 2 years

**Fig. 2:**
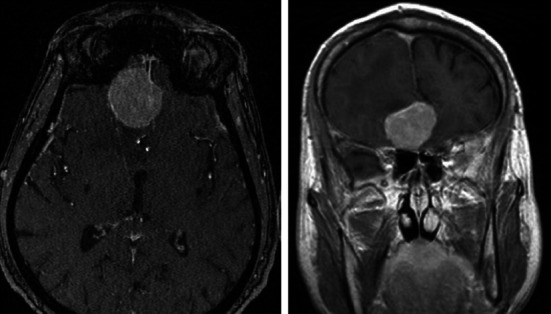
MRI (T2). Male patient aged 57—case number 2. History of anosmia and seizures for 2 years; personality distortion, worsening of verbal and logical contact on admission

**Fig. 3:**
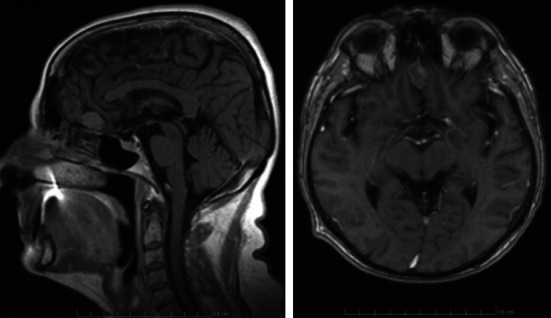
MRI (T2). Female patient aged 66—case number 3, history of anosmia for a year; vertigo, nausea and vomiting a week before admission

The records of three patients (two female, one male) treated by our team in 2011 and 2012 have been analyzed. The patients were 52, 57, 66 years of age, respectively. In each patient the leading symptom was unilateral anosmia observed within 1–2 years before the treatment. Two patients had a history of several episodes of seizures, one with concomitant verbal and logical contact deterioration. One patient presented with vertigo and nausea several days before the admission. However, none of the patients complained of very common in such a case oppressive headache. The diagnosis was based on CT and MR imaging (Figs. 1, 2, 3). All of the patients were qualified for surgical treatment by our otolaryngologist/neurosurgeon team.

### Surgical treatment

**Fig. 4:**
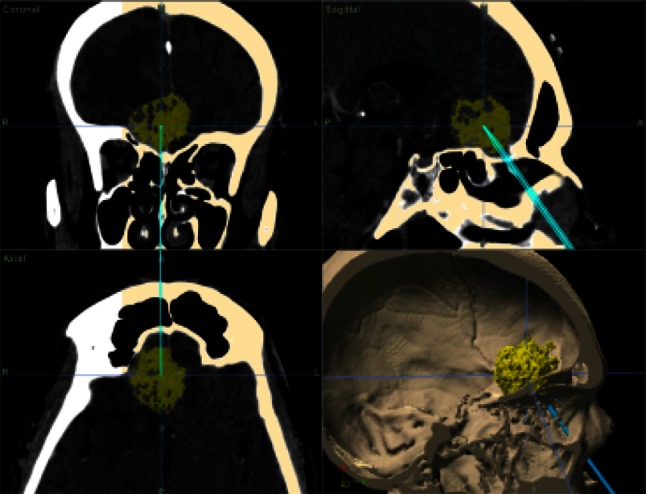
Neuronavigation—intraoperative image. Case 2

**Fig. 5:**
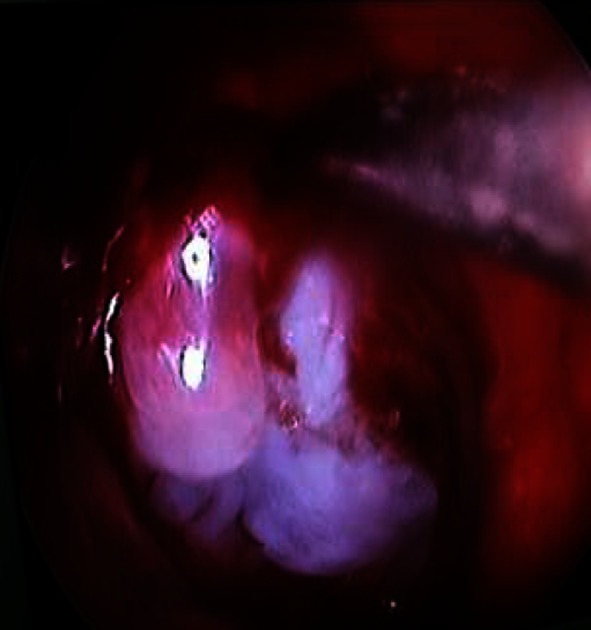
Intraoperative image. Tumour setting in the defect of the skull base

The procedure was performed under TIVA (total intravenous anaesthesia) with tracheal intubation on a patient in supine position with the head fixated and extended by 15°. During the whole operation controlled hypotension was induced with the mean blood pressure of 90 mmHg. The first step was local nasal mucosa decongestion with a solution of epinephrine (1:5,000). Then, using the endoscope with 0^o^ optics the cribrose palate was unveiled. In each case the middle turbinate was excised but nasal septum remained intact which later served for reconstruction. After visualization of the cribrose palate by means of neuronavigation the tumour was localized (Fig. 4) and mucosa of the palate was removed. At this stage of the procedure we used 0^o^, 30^o^ and 45^o^ endoscopes. The cribrose palate was milled with high-speed drill and then removed with Kerrison punches. After opening of the anterior cranial fossa we did not observe any significant cerebrospinal fluid (CSF) leakage due to the impression of the tumour in the defect in the skull base (Fig. 5). The diameter of the defect depended on the tumour size, yet it did not exceed 1–2 cm in the axial plane and 2–2.5 cm in horizontal plane apart from case number 1 when it sized 2.5 × 3 cm. The cut in the matrix was performed from the front to the rear and sized similarly to the defect in the skull base. This stage of the procedure was performed by the otolaryngologist. At the same time there were 3–4 tools inserted in the nose. In cases of a larger bleeding from the mucosa we stopped it with bipolar coagulation (bayonet or forceps). In all our patients with the assistance of neuronavigation we performed targeted approach to the cranial base (the mere point where the tumour was attached to the olfactory groove). Hence, there was no need for identification of the frontal, sphenoid sinus or Draff III procedure, especially that the tumours were relatively small.

The next stage of the operation was led by a neurosurgeon under a microscope using an automatic nasal retractor. Once the skull base was unveiled with the assistance of neuronavigation the centre of the tumour was identified and served as the target for drilling. Once the tumour was visualized, the neurosurgeon precisely assessed its margins, then separated it from the surrounding tissues, dissected from the matrix and removed it. Brain tissues remained intact. We tended to remove the tumour in one piece which was achieved in 2 out of 3 cases. In each case the resection was assessed as total. Haemostasis was achieved by means of bipolar diathermy. No significant bleeding was observed in all cases. The control of the postoperative cavity was performed endoscopically (30^o^, 45^o^ and 70^o^). Crista gali or falx cerebri were not approached during the operation. In the meantime a portion of the fat tissue, fragment of the fascia lata and quadriceps muscle was harvested. The reconstructive stage was performed by the otolaryngologist. The postoperative cavity was firstly filled with pieces of fat and muscle tissue, then with TachoSil and finally the fascia. We used tissue adhesive between the two layers. In the first patient, in which the defect in the skull base was the largest, for better reconstruction the titanium mesh was used. In the remaining two cases the “dense part” of the skull base was reconstructed with a fragment of the septal cartilage. Finally, the wound was covered with pedicled nasoseptal flap. In each case the multilayer reconstruction was performed according to the scheme: fat tissue-tissue adhesive-cartilage/mesh-fascia-tissue adhesive-nasoseptal flat-nasal packing. Tumour sizes and the reconstructive material are shown in the table.

**Table:** 

	Tumour size (cm)	Reconstructive material
Case 1	2.5 × 3	Titanium mesh
Case 2	1 × 2.5	Septal cartilage
Case 3	2 × 2	Septal cartilage

**Fig. 6:**
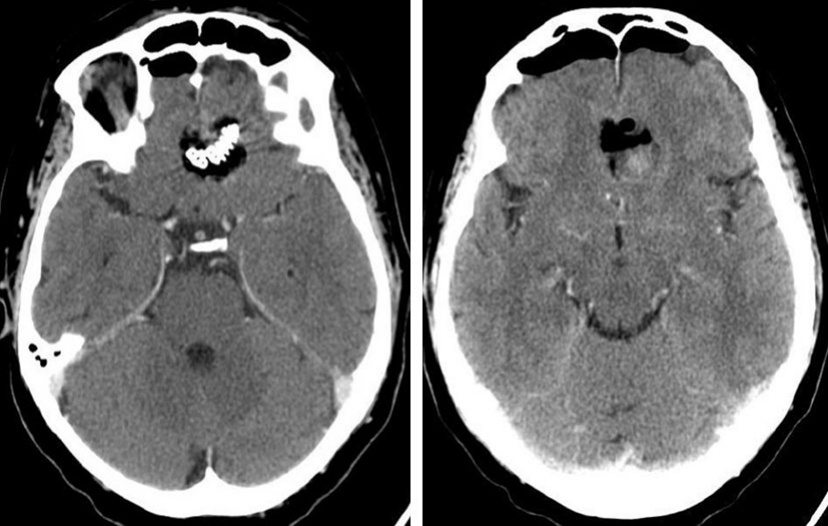
Case number 1. Follow up CT scan. Skull base reconstructed with a titanium mesh

**Fig. 7:**
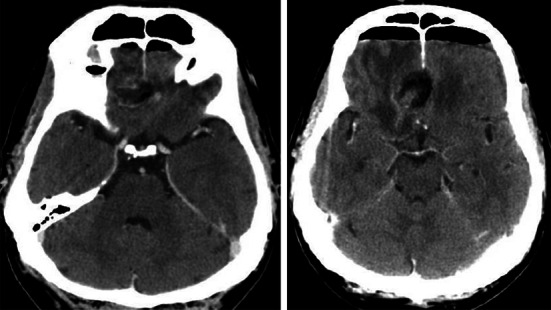
Case 2—follow up CT scan

**Fig. 8:**
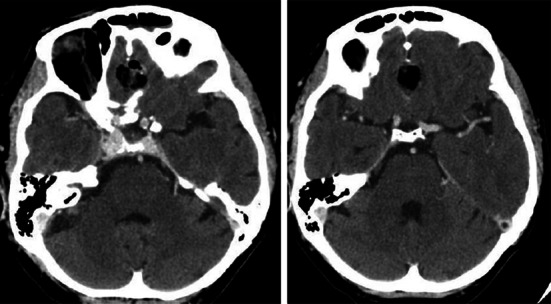
Case 3—follow-up CT scan

The procedure ended with the insertion of the nasal packing which was removed after 4–5 days. We decided to remove it as soon as possible, since it is very uncomfortable for the patient. All the operations were performed intranasally, unilaterally. Preoperatively standard prophylactic antibiotics and anti-oedematous drugs were administered. In each patients, we used lumbar drainage which was maintained for 3–4 days to reduce the risk of CSF leakage. The duration of the operation was 3 h 30 min, 5 h 30 min and 2 h and 15 min, respectively. No episode of CSF leakage was observed. In each patient 2–4 days after surgery the CT scan was performed (Figs. 6, 7, 8). Pathological examination revealed meningioma (WHO grade I). The hospitalization period lasted from 5 to 8 days. None of the patients suffered from meningitis in the perioperative, as well as, in postoperative observation period. For a few months after surgery we strongly recommended limitation of physical activity and introduced bowel regulation. Follow-up at 22, 12 and 8 months—no tumour recurrence.

## Discussion

### Otolaryngological part of the procedure—what can be offered to a neurosurgeon?

A fundamental condition contributing to a successful operation at this stage is scarce bleeding which can be achieved by mucosal decongestion with epinephrine solution and induction of controlled hypotension as in all our patients during functional endoscopic sinus surgery (FESS). In order to visualize the base of anterior cranial fossa (olfactory groove), as well as, the sellar region we considered it vital to resect the middle turbinate. However, contrary to the approach via sphenoid bone to the olfactory groove, it is not necessary to remove the posterior part of the nasal septum [3, 10]. Lack of manipulation on the lower turbinate diminishes the risk of postoperative empty nose syndrome. In addition, careful and atraumatic approach to the lower turbinate guarantees absence of nasal adhesions and deterioration of nasal function in the late postoperative period. This complication did not occur in our patients which is concordant with other reports [2]. Bone thickness of the anterior cranial fossa base in otherwise healthy patients (with no history of trauma or sino-nasal operations) usually reaches several millimetres which impose the use of high-speed drill with adequate cooling mechanism (three or four hands technique) [10]. Other than TNE approach to this site seems to be, in our opinion, less secure. Recommended by van Gompel et al. defect size in the skull base (15–20 mm × 25–30 mm) depends on the tumour size. However, the use of neuronavigation allows to minimize the defect to rational boundaries [9]. The opening of the anterior cranial fossa through the posterior frontal sinus wall and sella turcica is being performed by other surgeons [1, 2]. Nevertheless, a reconstruction of the osteodural defect in such a case poses a difficult challenge and, therefore, the risk of brain herniation developing in the postoperative period is highly increased [10, 11]. Apart from the first case, in the remaining two the bony defect size varied from 10–20 to 20–25 mm. The mere tumour removal and, above all, postoperative cavity control is very precise when using 30^o^ and 45^o^ endoscopes [2, 4, 10].

### Tumour resection: neurosurgical part of the procedure

A crucial condition of the total resection is the removal of the starting point of the tumour. Many authors emphasize that in all of the patients the aim of the operation remains total or near-total resection (>95 %) of the tumour [1, 2, 11]. In the material of Gardner et al. total removal [13] was not achieved in 2 cases out of 15 due to significant bleeding and poor visualization. Van Gompel et al. in their meta analysis describing cases of treatment of the anterior cranial fossa meningiomas (olfactory groove and sella turcica region) report that total resection constituted 88 % of the cases [1, 9]. Complete excision and, thus, decrease in the risk of recurrence is directly associated with the resection of the anterior fossa floor [2]. To achieve this goal, Obeid et al. [12] advice to drill the hyperostotic bone of the groove when external approach is applied. Endoscopic opening of the groove entails vast bone resection including in some cases part of the matrix which significantly improves the results [10]. In each of our patients we tended to perform type I resection according to Simpson (tumour, matrix and bone) which according to many authors reduces the incidence of recurrence to 9–15 % [1, 4, 11]. CSF leakage represents a crucial issue in TNE approach. The percentage of patients suffering from this complication after olfactory groove meningioma resection varies from 0 to 40 % or even up to 65 % in earlier reports and is basically determined by surgeon’s degree of experience. However, introduction of modern reconstructive techniques diminished the problem to 5.4 % of the cases [1, 8].

### Skull base reconstruction: otolaryngological assignment

The defect in the skull base (usually sized 2–5 cm^2^) requires multilayer closure. In the first stage, the cavity after resection was filled with fat and muscle tissue and fragment of fascia lata using underlay method (between the matrix and the bone). The packing was then joined with adhesive tissue. Secondly, a fragment of nasal cartilage or a titanium mesh in one case, was placed superficially (case number 1—the aperture size exceeded 7.5 cm^2^). Finally, the wound was covered with a TachoSil (CSL Behring GmbH, Germany) and a vascularised mucosal flap. Introduction of local pedicled flap based on the posterior vessels of the nasal septum containing mucoperiosteum and mucoperichondrium (Hadad-Bassagasteguy flap) significantly improved robustness of the multilayer bung [2, 4–6, 11]. The flat (the last layer of the reconstruction) is usually placed overlay [1, 11] and supported with anterior nasal packing. For improved sealing efficiency in all our patients one of the layers consisted of a rigid element (nasal septum cartilage or titanium mesh). The operation was finished with nasal packing which was removed after 4–5 days. Such an early removal of the packing (when compared to other authors) was possible due to multilayer and thus secure method of reconstruction [2]. In each our patient the lumbar drainage was used and maintained for 4 days which is consistent with other authors [2], although Oostra et al. [4] reserved this procedure for a recurrent CSF leakage. We would like to emphasize that in none of our patients CSF leakage was observed in postoperative period.

## Advantages of endoscopy: summary

Transnasal endoscopic approach constantly gains popularity as a feasible and effective therapeutic method for olfactory groove meningioma. Up to date there have been 30 reported cases in available literature [1, 2, 4, 7, 10, 14]. Endoscopic approach to the anterior cranial fossa lesions assures not only absence of a visible scar (acceptable cosmetic outcome) but also obviated brain retraction and neurovascular structures manipulation. Introduction of neuronavigation allows better localization of important structures facilitating targeted resection. TNE technique enables direct and quick access to the tumour with creating a natural way to remove it. Endoscopy facilitates good visualization and, hence, secure and complete resection of its dural and bony origin. This method can be accomplished with reasonable morbidity rate. Furthermore, the risk of some major neurological complications (including haematoma, damage to contiguous structures), according to some authors (de Divitiis et al.), can be lower when compared to open methods [1, 2, 10]. Shorter duration of the operation and hospitalization contributes to quicker recovery and cost reduction of the whole treatment.
